# The interval approach: an adaptation of the liver-first approach to treat synchronous liver metastases from rectal cancer

**DOI:** 10.1186/s12957-017-1123-6

**Published:** 2017-03-02

**Authors:** Mathieu D’Hondt, Valerio Lucidi, Koen Vermeiren, Bert Van Den Bossche, Vincent Donckier, Gregory Sergeant

**Affiliations:** 1 Department of Abdominal Surgery, AZ Groeninge, Kortrijk, Belgium; 2 Department of Abdominal Surgery, Hôpital Erasme, Brussels, Belgium; 3 Department of Abdominal Surgery, AZ Imelda, Bonheiden, Belgium; 4 Department of Abdominal Surgery, Algemeen Stedelijk Ziekenhuis, Aalst, Belgium; 5 Department of Abdominal Surgery, Jessa Ziekenhuis, Salvatorstraat 20, B-3500 Hasselt, Belgium; 6 Faculty of Medicine and Life Sciences, UHasselt, Hasselt, Belgium

**Keywords:** Synchronous liver metastasis, Rectal cancer, Liver resection, Simultaneous approach, Delayed approach, Interval approach

## Abstract

**Background:**

The waiting interval after chemoradiotherapy (CRT) is an interesting therapeutic window to treat patients with synchronous liver metastases (SLM) from rectal cancer.

**Methods:**

A retrospective analysis was performed of 18 consecutive patients (M/F 10/8, age (range) 60 (51–75) years) from five institutions who underwent liver resection of SLM during the waiting interval after CRT for rectal adenocarcinoma.

**Results:**

All patients underwent interval liver surgery for a median (range) of 4 (2–14) liver metastases. Metastases involved a median (range) of 4 (1–7) liver segments. Median (range) time between end of CRT and liver surgery was 22 (6–45) days. Laparoscopic liver surgery was performed in 12 (67%) patients. No severe complications (Clavien-Dindo ≥ 3b) occurred after liver surgery. Median (range) length of hospital stay after liver surgery was 5 (1–10) days. All patients subsequently underwent rectal resection at a median (range) of 10 (8–13) weeks after end of CRT. Median (IQR) time-to-progression after liver surgery was 4.2 (2.8–9.2) months.

**Conclusions:**

The waiting interval after neoadjuvant CRT is a valuable option to treat SLM from rectal cancer. More data are necessary to confirm its oncological efficacy.

## Background

Patients with colorectal cancer develop liver metastases in 60% of cases during the course of their disease. In 15–25% of patients with colorectal cancer liver metastases are present at time of initial diagnosis, i.e., synchronous liver metastases [1]. In this group of patients, several strategies have been proposed to manage both primary and liver metastases. These strategies may be categorized as either a delayed approach, sometimes also referred to as staged approach, or a simultaneous (i.e., combined) approach in which colorectal resection and liver surgery are performed during a single anesthesia [2]. The delayed approach may be further divided in a colon-first or liver-first approach [3, 4] depending on which organ is operated first. Which strategy to choose depends on the presentation of the primary tumor and extent of liver disease. Especially in advanced or borderline resectable liver metastatic disease, it may be preferable to first eradicate liver metastases after a preoperative course of (conversion) chemotherapy.

A recent meta-analysis combining data on 18 studies and 3605 patients with synchronous colorectal liver metastasis did not demonstrate a clear statistical surgical outcome or survival advantage of any of these three approaches [5]. Meta-analysis of these non-randomized trials is problematic, as a major selection bias exists. Indeed, it has previously been found that patients undergoing colon-first or simultaneous approach had less advanced liver disease. Also, patients with rectal cancer represent only a small subgroup within this trial population and it is unknown whether the same conclusions may be drawn for these patients. The management of patients with synchronous liver metastases from rectal cancer is further complicated by the need for neoadjuvant chemoradiotherapy in those cT3 + N0/+ cancers arising in the lower two thirds of the rectum. Also, a simultaneous approach is generally not advocated for patients with simultaneous liver metastases from rectal cancer, owing to an increased risk for postoperative complications [6, 7].

As there is a trend to increase the waiting interval up to 10 weeks after neoadjuvant chemoradiotherapy (CRT) for rectal adenocarcinoma to optimize treatment response [8], an interesting therapeutic window has opened up to treat patients with synchronous liver metastases. Our aim was to perform a multi-institutional analysis of patients with rectal cancer who underwent interval laparoscopic liver resection of synchronous colorectal liver metastases.

## Methods

Between 2010 and 2015, 18 patients (M/F 10/8, age (range) 60 (51–75) years) at 5 different Belgian institutions (non-academic teaching hospital (*n* = 4) and academic hospital (*n* = 1)) underwent liver surgery during the waiting interval after CRT for rectal adenocarcinoma presenting with synchronous liver metastases.

Demographic, tumor-related, surgical procedure-related, and outcome parameters were retrieved from this database. Complications were registered and categorized according to the Clavien-Dindo [9] and the Comprehensive Comorbidity Index (CCI) [10].

Survival analysis was performed using the common closing date for follow-up. Median (range) follow-up after liver surgery was 20.5 (3.6–63.1) months. Time-to-progression and overall survival was calculated using Kaplan-Meier survival plots.

Ethical approval was obtained from the institution coordinating the retrospective study (Belgian registration No: B243201524173).

## Results

All patients underwent interval liver surgery for a median (range) of 4 (2–14) liver metastases. Liver metastases involved a median (range) of 4 (1–7) liver segments. Portal vein embolization was performed prior to liver resection in 5 patients. Median (range) time (days) between last irradiation and liver surgery was 22 (6–45) days. Laparoscopic liver surgery was performed in 12 (67%) patients (10 resections, 2 radiofrequency ablations). Types of liver resections are summarized in Table 1. Median (range) of operating time was 186 (100–450) min.

**Table 1:** Type of liver resection with location of liver metastatic disease for minor hepatectomies

Type of liver resection (Brisbane-classification)	Segments	*N* patients
Right hemihepatectomy	5–8	6
Extended left hemihepatectomy	1–5, 8	1
(Bi)segmentectomy	Pt 2 (2, 3) Pt 11 (7) Pt 12 (8) Pt 16 (2, 4, 6) Pt 17 (6–8)	5
Metastasectomy	Pt 4 (6, 8), Pt 8 (2–5, 7, 8), Pt 13 (3–5, 7), Pt 14 (7, 8)	4
Radiofrequency ablation	Pt 7 (6, 8)Pt 10 (6, 7)	2

Five patients developed a postoperative complication after liver surgery. One patient developed biliary fistula requiring surgical intervention under general anesthesia (Clavien-Dindo ≥ 3b). Median (range) CCI was 0 (0–33.7). Median (range) length of hospital stay after liver surgery was 5 (1–10) days. All patients subsequently underwent rectal resection without treatment delay at a median (range) of 10 (8–13) weeks after the last irradiation. Rectal resections consisted of two partial mesorectal excisions (PME), 14 total mesorectal excisions (TME), and two abdomino-perineal rectal amputations (APR). Rectal resections were performed laparoscopically in 15 patients.

Ten patients developed tumor progression during follow-up. Median (interquartile range) time-to-progression after liver surgery was 4.2 (2.8–9.2) months. The liver was one of the sites of tumor progression in 7/10 patients. Three patients died during follow-up. The median overall survival in our cohort could not be calculated because after a median follow-up time of 20.5 (3.6–63.1) months, more than 50% of patients were still alive.

## Discussion

To our knowledge, this is the first report of using the waiting interval after CRT for resection of synchronous liver metastases from rectal cancer. In addition, we show that by using a laparoscopic approach for both liver and rectal surgery, this may be achieved with minimal morbidity and short hospital stay.

Insufficient evidence is available to guide the precise extent of liver resection that can be safely undertaken in combination with colorectal resection [11]. “Delayed” resections are favored over simultaneous resections in patients with synchronous colorectal liver metastasis when extensive liver disease (≥3 segments) [12]. This may be one of many other reasons why the *simultaneous* approach has not found many enthusiasts. In addition longer operating times (>300 min), the increased technicity of both liver and rectal resections, practical issues regarding operation room setup (a fortiori for laparoscopic surgery) [2], and the high postoperative morbidity for major liver resections [13] have limited the widespread adoption of the simultaneous approach especially for treatment of synchronous liver metastatic disease from rectal adenocarcinoma.

*Simultaneous* resection for rectal cancer might be associated with more postoperative complications [6]. Moreover, oncological concerns have been expressed against the simultaneous approach. LiverMetSurvey analysis showed significantly worse overall and progression-free survival rates at 3 years for the simultaneous approach compared to the *delayed* group [14].

Therefore, our approach of using the waiting interval for liver resection is extremely tempting as it may provide the solution to avoid an increased risk of complications and mortality when performing liver and rectal resection during the same anesthesia. The interval approach is a variation of the *delayed* approach, without lengthening the overall treatment duration. Interestingly, despite the fact that all participating centers in the retrospective cohort share a parenchyma-sparing approach to liver surgery for colorectal liver metastase, the majority of patients underwent an extensive hepatic resection. It is however known that patients presenting with synchronous liver metastases have more advanced disease (i.e., more metastases and more often bilobarly distributed metastases) at presentation [15]. This fact may at least partially explain why most of the patients in this cohort underwent extensive resections. Also, some bias may exist as in the participating centers for patients with limited metastatic disease eligible for a limited number of (laparoscopic) liver-sparing resections a simultaneous approach would have been favored. We are aware that complications after liver surgery (especially major liver resection) are not uncommon. Review of the literature learns that complications occur at a frequency of 5–15% for minor laparoscopic resections to up to 50% for major open resections. It is therefore the policy of participating centers in patients with multiple or large liver metastases or where a major liver resection is anticipated for R0 resection, to start with upfront chemotherapy in order to obtain a good hepatic and extra-hepatic systemic control. Following this chemotherapy, a radiological re-evaluation is executed. In case of an objective radiological response, chemoradiotherapy may be initiated and liver resection can be performed during the waiting interval. In those cases where the planned liver resection would be a trisectionectomy or a procedure necessitating vascular or biliary reconstruction, we would not perform liver resection during the waiting interval after chemoradiotherapy, but rather preceding chemoradiotherapy.

To our surprise, median time to tumor progression in our cohort was only 4.2 months. The liver was among the primary site of tumor progression in 7 out of 10 patients. This finding may reflect the advanced stage of disease at diagnosis or at least its aggressive tumor biology. A similar conclusion was drawn recently by Welsh et al. [16] that patients with simultaneous colorectal liver metastases selected for a liver-first approach had more advanced disease and a poorer prognosis. These patients had a inferior cumulative disease-free survival than those patients undergoing a classical approach, a difference negated by matching preoperative Basingstoke Predictive Index [16].

Indeed, as an example of advanced disease in our cohort, resection specimens of eight patients revealed at best a yN2a nodal status after CRT. Nevertheless, after a median follow-time of 20.5 months, the median overall survival could not be estimated. Another explanation for the early tumor progression seen in our cohort may be that selection of patients with excellent prognosis may be more difficult in patients with synchronous liver metastases, especially when the interval approach is used.

We are aware of some shortcomings of our study. All together, this remains a retrospective study with relatively small sample size. The small sample size precludes in-depth analysis of other prognostic factors (e.g., preoperative CEA levels) to explain the early tumor progression seen in our series. In order to increase overall sample size, we have merged experience of different centers.

Thereby, we have showed that this approach is not exclusive to just one center (single-center experience) but has succesfully been used in different centers in selected cases with low morbidity both for a laparoscopic and open approach. Long-term prospective studies with overall and disease-free survival are needed to confirm its oncological non-inferiority compared to the other conventional approaches. A randomized controlled trial avoiding selection bias and looking at time-to-progression as a primary endpoint may be the ideal tool for this.

## Conclusions

The waiting interval after neoadjuvant CRT seems a valuable option to treat synchronous liver metastases from rectal cancer with an acceptable safety profile. Many of the drawbacks from classical delayed approaches may be overcome without lengthening the overall treatment duration. More prospective long-term follow-up data are necessary to confirm its oncological efficacy.

## Abbreviations

5-FU: 5-Fluorouracil
CCI: Comprehensive complication index
ce: Contrast-enhanced
CRT: Chemoradiotherapy
